# A268 RESOLUTION RATES IN OPEN-LABEL VERSUS RANDOMIZED CONTROLLED TRIALS FOR MICROBIOTA RESTORATION FOR RECURRENT CLOSTRIDIOIDES DIFFICILE INFECTION: AN UPDATED META-ANALYSIS

**DOI:** 10.1093/jcag/gwac036.268

**Published:** 2023-03-07

**Authors:** R Tariq, D S Pardi, S Khanna

**Affiliations:** Mayo Clinic, Rochester, United States

## Abstract

**Background:**

Microbiota restoration is highly effective to treat recurrent *Clostridioides difficile* infection (CDI) in observational studies (cure rates >90%) but efficacy in controlled clinical trials appears lower.

**Purpose:**

We performed an updated meta-analysis to assess the efficacy of microbiota restoration for recurrent CDI in open-label registered prospective clinical trials compared to randomized controlled trials (RCTs).

**Method:**

A systematic search of Embase, Web of Science and Scopus was performed up to June 2022 to identify studies of interest. Clinical trials of microbiota restoration for recurrent CDI with clinical resolution with one dose as the primary outcome were included. We calculated both unweighted and weighted pooled resolution rates (UPR and WPR) with 95% confidence intervals (CI).

**Result(s):**

Eighteen studies (9 RCTs and 9 open-label trials) with 1149 CDI patients were included. Of the patients treated with microbiota restoration, 881 experienced symptom resolution (UPR 77%%; WPR 79%, 95% CI, 72%-85%). There was significant heterogeneity among studies with an *I*^*2*^of 86%. Analysis of trials with a control arm (non-microbiota restoration) revealed CDI resolution in 357 of 496 patients (UPR 72%; WPR 73%, 95% CI 63%-82%) with microbiota restoration. Among the 9 open-label clinical trials, CDI resolution was seen in 524 of 653 patients after initial microbiota restoration (UPR 80%; WPR 84%, 95% CI 74%-92%). Comparison of resolution rates between RCTs and open-label trials revealed a lower cure rate in RCTs compared to open-label trials (WPR 73% vs 84%, p<0.0001).

Analysis of the 10 trials with non-microbiota restoration revealed CDI resolution in 201 of 397 patients with antibiotics (WPR 52%, 95% CI 43%-60%). There was significant heterogeneity among the included studies with an *I*^*2*^of 61%. Comparison of cure rates with microbiota restoration vs antibiotics showed higher cure rate with microbiota restoration (WPR 73%, [95% CI 63%-82%] vs 52% [95% CI, 43%-60%]; p<0.0001). There were no serious adverse events reported.

**Conclusion(s):**

Microbiota restoration in a randomized controlled setting leads to lower resolution rates compared to open label and observational settings, likely due to stricter definitions and inclusion criteria. Resolution rates in open label studies were similar to observational studies.

**Please acknowledge all funding agencies by checking the applicable boxes below:**

None

**Disclosure of Interest:**

None Declared

